# Risky Behaviors for Non-Communicable Diseases: Italian Adolescents’ Food Habits and Physical Activity

**DOI:** 10.3390/nu16234162

**Published:** 2024-11-30

**Authors:** Gaia D’Antonio, Vincenza Sansone, Mario Postiglione, Gaia Battista, Francesca Gallè, Concetta Paola Pelullo, Gabriella Di Giuseppe

**Affiliations:** 1 Department of Experimental Medicine, University of Campania “Luigi Vanvitelli”, 80138 Naples, Italy; gaia.dantonio@unicampania.it (G.D.); vincenza.sansone@unicampania.it (V.S.); mario.postiglione@unicampania.it (M.P.); gaia.battista@unicampania.it (G.B.); gabriella.digiuseppe@unicampania.it (G.D.G.); 2 Department of Medical, Movement, and Well-Being Sciences, University of Naples “Parthenope”, 80133 Naples, Italy; francesca.galle@uniparthenope.it

**Keywords:** adolescent, food habits, healthy eating, physical activity, risky behaviors

## Abstract

Background: Driving adolescents to more correct food habits and physical activity is crucial to promoting health and avoiding the increase in morbidity and mortality in adulthood. Literature has focused on these behaviors in the adult population, while studies on adolescents are more limited. This study aims to explore the level of knowledge, attitudes, and behaviors regarding nutrition and physical activity to acquire insight into adolescents and identify the associated predictors. Methods: A cross-sectional study was conducted among adolescents aged 10 to 19 years from public middle and high schools randomly selected in the Campania Region, Southern Italy. A self-administered questionnaire, including closed and open-ended questions, assessed socio-demographic and health-related characteristics, dietary habits, physical activity, and sources of health information. Results: Regarding socio-demographic and health-related characteristics, among 1433 adolescents who completed the survey, the mean age was 15.2 years, 50.5% were boys, 16.8% reported having a non-communicable disease, and 18% were overweight or obese. Multivariate analysis showed that older age, male gender, daily breakfast with at least one parent, higher self-rated knowledge on nutrition, awareness of fruit and vegetables consumption recommendations, correct dietary attitudes (daily breakfast, consumption of fruit and vegetables at least once a day, of legumes at least twice a week, and of carbonated sugary drinks less than once a day), the need for additional dietary information, meeting WHO physical activity recommendations, and less than two hours of daily screen time are determinants of a high quality diet score. Conversely, living with a single family member and current smoking were negatively associated with high quality diet. Older age, male gender, risk of alcohol abuse, higher quality diet, and lower mobile phone use are associated with meeting WHO physical activity recommendations. Since we investigated risky behaviors, potential limitations of this study could include social desirability and recall bias. Conclusions: Many adolescents lead unhealthy lifestyles, but younger adolescents and girls appear to be at higher risk of unhealthy behaviors. Targeted initiatives promoting regular physical activity and balanced diets in schools, involving parents and teachers in a collaborative plan, are essential to improving adolescents’ health and well-being.

## 1. Introduction

Non-communicable diseases (NCDs), including cardiovascular disease, cancer, diabetes, and obstructive lung diseases, are responsible for almost three-quarters of deaths in the world each year. Worldwide, millions of people have at least one NCD, which affects their quality of life [1]. Around 24 million individuals in Italy are affected by NCDs, which have an important role in the overall quality of life and life expectancy of the population. These diseases can affect individuals of all ages, but they are more prevalent among the elderly, with over 85% of those older than 75 being affected, and women, especially those over 55, as they enter menopause and may begin gaining weight [2]. NCDs are one of the global goals of the WHO 2030 agenda that aims to reduce premature mortality from these diseases by one-third through prevention and treatment. This objective is also present in the Italian National Prevention Plan 2022–2025, which encourages a life course approach that reduces individual risk factors and removes the causes that prevent citizens from accessing healthy environments and lifestyles [3].

Many NCDs are often preventable by eliminating risks to health and, in particular, behaviors such as tobacco use, unhealthy diet, harmful use of alcohol, and physical inactivity [4,5,6]. These determinants during adolescence establish patterns of behavior that can directly influence body image perception and NCDs in adulthood [7,8,9,10,11,12,13,14]. Indeed, adolescence is a critical period when lifelong behaviors are established, influencing NCD risk in adulthood and shaping health outcomes for individuals and communities [15,16,17,18]. In particular, healthy eating behaviors and engaging in regular physical activity (PA) are essential for the well-being of adolescents, as overweight and obesity at this stage of life can lead to NCDs in adulthood [8,9,10]. Therefore, the evaluation of knowledge, attitudes, and behaviors of adolescents regarding nutrition and PA could represent an opportunity to promote healthier lifestyle choices and reduce NCD risk. This evaluation is essential to understand which public health program should be promoted to avoid increasing morbidity and mortality in adulthood [19,20,21,22,23].

In the literature, several studies investigating knowledge, attitudes, and behaviors about healthy eating and PA have been conducted in the adult population [24,25,26,27], while evidence on adolescents is more limited to different food intake, body conception, or sedentary lifestyle [28,29,30]. Thus, the present survey aims to explore the level of knowledge, attitudes, and behaviors regarding nutrition and PA in adolescents aged 10–19 in Southern Italy and to identify socio-demographic characteristics and other lifestyles associated with the outcomes of interest.

## 2. Materials and Methods

### 2.1. Setting and Participants

This cross-sectional study was part of a larger project about adolescents’ lifestyles and perceptions of climate change based on the One-Health approach [3]. This study was conducted—from February to April 2024—among 1437 students aged 10 to 19 years attending public middle (from 10 to 13 years old) and high schools (>13 years old) in the geographical area of the Campania region, Southern Italy. The sample was selected through a two-stage cluster sampling procedure. In the first stage, four middle schools and five high schools were randomly selected from a list of regional public schools. In the second stage, adolescents were selected from each school using a simple random technique.

### 2.2. Sample Size

The sample size was determined before study initiation, considering a 95% confidence level, a margin of error of 5%, a design effect of 2, and a response rate of 50%. Since previous data were not homogeneous, adolescents that have healthy eating and PA were assumed in 50% of students [31]. Therefore, the minimum sample size was estimated to be 768 participants. In order to increase the precision of the results, a larger sample of adolescents was recruited.

### 2.3. Data Collection

Before starting the survey, the directors of each randomly selected school were first contacted to arrange an informative meeting, during which they received a letter outlining the project objectives and methods for data collection. After the approval, the students were given a sealed envelope to take home that contained a cover letter about the purpose and objectives of the survey, as well as an informed consent form. For adolescents under 18 years of age, voluntary participation in this study required authorization from their parents or legal guardians. Privacy and anonymity were strictly ensured, and this policy was stated on the front page of the questionnaire. Ensuring that consent had been acquired, the questionnaire was distributed to students in their classroom, where a trained researcher provided verbal instructions for accurate completion. Moreover, the importance of providing honest responses was emphasized, with an explanation that data would be aggregated to protect anonymity. A member of the research team was in the classroom to help students if any questions were not clear or for any doubts. No payments or gifts were given to the respondents.

### 2.4. Survey Instrument

The self-administered structured questionnaire included closed and open-ended types of questions. Open-ended questions have been codified by the research team, and all responses have been analyzed using categorical variables. The questionnaire consists of three sections:

1. Adolescents’ socio-demographic and health-related characteristics (age, gender, nationality, parents’ education level, and parents’ occupation, having a NCD, alcohol use, smoking habits, weight, and height to obtain BMI-for-age percentile based on CDC growth charts for children and teens ages 2 through 19 years, etc.) [32]. The self-perceived health status was measured on a Likert-type scale ranging from 1 (not at all satisfied) to 10 (very much satisfied).
2. Adolescents’ dietary habits. The section on dietary habits was created ad hoc based on previously used tools in the literature, and it was divided into sections as follows: (a) knowledge towards recommended consumption of different foods (fruit, vegetables, meat, fish, etc.) using true/false and multiple choice closed questions; self-rating of healthy dietary habits knowledge using a 1 to 10 Likert-type scale; (b) attitudes about nutrition using a 3-point Likert-type scale with agree/uncertain/disagree response format; (c) sources of information on diet and need to receive additional information about dietary habits; (d) behaviors such as breakfast frequency, meals with parents and daily consumption of healthy or unhealthy food were taken from the Food Frequency Questionnaire (FFQ), a module in the Health Behaviour in School-Aged Children (HBSC) questionnaire [33,34,35]. Students were asked: “On how many times a week do you usually eat/drink (food/beverage)?”. The options were: “never”, “less than once a week”, “once a week”, “2–4 days a week”, “5–6 days a week”, “once every day”, and “several times every day”. A diet quality score was assessed using five indicators: frequency of schooldays breakfast, consumption of fruit, vegetables, legumes, and carbonated sugary drinks. Using the above five dietary indicators, a global score was created by counting the number of correct habits, ranging from 0 to 5: daily breakfast (no = 0; yes = 1), consumption of fruit at least once a day (no = 0; yes = 1), consumption of vegetables at least once a day (no = 0; yes = 1), consumption of legumes at least twice a week (no = 0; yes = 1), and consumption of carbonated sugary drinks less than once a day (yes = 0; no = 1); this global score was dichotomized into “less than 3” (poor quality diet) vs. “at least 3” (good quality diet) [36]. Furthermore, questions concerning daily water intake and salt use were added.
3. Adolescents’ PA. (a) PA levels were assessed using the International Physical Activity Questionnaire-Short Form (IPAQ-SF) [37,38]. The seven items of IPAQ-SF assessed the total energy expenditure per week by considering the number of days and minutes spent on vigorous PA (8 Metabolic Equivalents-METs), moderate PA (4 METs), and walking (3.3 METs). The IPAQ total score is expressed in MET-minutes/week and represents an index of inactivity (<700 MET-minutes/week), sufficient activity (700-2519 MET-minutes/week), or high activity (>2520 MET-minutes/week). Furthermore, adolescents’ PA was also dichotomized into meeting or not meeting World Health Organization (WHO) recommendations. WHO recommends that children and adolescents aged 5–17 years should accumulate at least an average of 60 min per day of moderate-to-vigorous-intensity PA at least 3 days a week, whereas people who are 18 years or older should accumulate at least 150 min of moderate-intensity PA or at least 75 min of vigorous-intensity PA per week, or an equivalent combination [5,39,40]. (b) Questions about screen time and phone usage in the past week [36,41] and the importance of engaging in sports using a 1 to 10 scale. (c) Adolescents’ ideal body image questions were taken from the School Physical Activity and Nutrition (SPAN) questionnaire [42,43]. Three questions regarding current appearance (current silhouette) and desired appearance (ideal silhouette) were asked. For the first two questions concerning the ideal silhouettes, the figures were numbered from 1 to 7 for boys and from 8 to 14 for girls, representing ideal conditions from extreme thinness to obesity; for the third question about adolescents’ current appearance, both boys’ and girls’ figures ranged from 1 to 14. Students were first instructed to indicate their current appearance and then their ideal appearance. A body image dissatisfaction index was obtained through the following calculation: current silhouette-ideal silhouette. Adolescents with positive values in this calculation were classified in the “want to lose weight” category, those with negative values were classified in the “want to gain weight” category, and those with zero values were classified in the “satisfied with their appearance” category. Then, the three categories were dichotomized in “satisfied” vs. “unsatisfied” with their appearance. (d) Source of information on PA and need of information.

A pilot study was conducted among 50 adolescents to evaluate the readability, clarity, and correct sequence of the questionnaire items. No changes have been made after the pilot study since all items were understandable and clear for the adolescents.

### 2.5. Ethics

This study protocol was approved by the Ethics Committee of the Teaching Hospital of the University of Campania “Luigi Vanvitelli” (approval number 0018199/i/01.07.2024). All sensitive, personal, and psychological data were treated according to the guidelines of the Declaration of Helsinki.

### 2.6. Statistical Analysis

All analyses were performed with the Stata software, version 17 [44]. First, descriptive statistics were conducted to summarize the main characteristics of the sample by using frequency and proportion for the categorical variables and mean (±standard deviation) for the continuous variables. Second, the Chi-square test, Chi-square for trend, and the Student *t*-test were calculated to identify determinants associated with outcomes of interest. The statistical significance level in the univariate test was set at *p* ≤ 0.25, according to Hosmer and Lemeshow [45]. Then, the independent variables reporting a *p*-value ≤ 0.25 in the univariate analysis were included in the multivariate logistic regression models to verify the significant relationship between dependent and independent variables. The significance level of the *p*-value for the inclusion and elimination of the variables in the models was set at 0.2 and 0.4, respectively. The two outcomes were: quality of diet score (Model 1), which was recorded as 1 if the score was “good diet quality” and 0 if it was “poor diet quality”, and met the WHO PA recommendations (Model 2), which was recorded as 1 if the WHO PA recommendations were met and 0 if they were not. The following independent variables have been tested for both models: age group (≤13 years = 0; >13 years = 1), gender (female = 0; male = 1), family members (single member = 0; more than one = 1), smoking habits (categorical) (never = 1; former = 2; current = 3). In the first model, the following independent variables were also added: parents’ education level (other = 0; at least one parent with a university degree = 1), breakfast with at least one parent (no = 0; yes = 1), lunch and dinner with at least one parent (no = 0; yes = 1), self-rating of healthy dietary habits knowledge (continuous), knowledge about recommended daily fruit and vegetables portions (incorrect = 0; correct = 1), dietary attitudes (incorrect = 0; correct = 1), sources of dietary information (none = 0; at least one = 1), need to receive additional information about dietary habits (no = 0; yes = 1), meeting the WHO PA recommendations (no = 0; yes = 1), hours spent per day watching TV or playing video games (≥2 h = 0; <2 h = 1), hours per day spent on the phone (≥2 h = 0; <2 h = 1). In the second model, the following independent variables were added: NCDs (no = 0; at least one = 1), being at risk of alcohol abuse (no = 0; yes = 1), sources of PA information (none = 0; at least one = 1), high quality diet score (no = 0; yes = 1), hours per day spent on the phone (continuous). Results are presented as Odds Ratios (ORs), and 95% Confidence Intervals (CIs) were calculated. All reported *p*-values are two-tailed, and a *p*-value ≤ 0.05 is considered statistically significant.

## 3. Results

### 3.1. Adolescents’ Socio-Demographic and Health-Related Characteristics

Of the 1875 eligible participants, 1437 completed the questionnaire. However, four participants were excluded for reporting an age > 19 years, leaving a total sample of 1433. The response rate was 76.6%. Table 1 displays an overview of adolescents’ socio-demographic and health-related characteristics.

The mean age was 15.2 years (range 11–19), of which most were Italians (96.8%), and approximately half of the sample was composed of males (50.5%). A total of 744 participants (51.9%) had at least one parent who had a university degree, and the vast majority, 95.2%, had at least one employed parent. Additionally, 96.6% lived with more than one family member. Regarding weight status, 77.3% of the sample was classified as having a healthy weight, while 4.7% were underweight, and 18% were overweight/obese. Moreover, 16.8% of participants had at least one NCD, such as respiratory diseases (67.3%), cardiac diseases (2%), and diabetes (1.6%), of which slightly more than half (53.5%) took medication.

Regarding parents’ NCDs, 20.8% of them referred to having at least one.

Respondents reported that their self-perceived health status, measured on a 10-point Likert-type scale, had an average of 8.1. Concerning smoking and alcohol consumption, 915 adolescents (65.1%) had never smoked, and 19.4% and 15.5% were former and current smokers, respectively. Additionally, 44.3% of adolescents were at risk of alcohol abuse.

### 3.2. Adolescents’ Dietary Habits

Many adolescents did not know the recommendations for fruit and vegetables daily intake (72.1%), but 91.8% understood the importance of fibers during digestion. When asked about their knowledge of the recommended weekly consumption of meat, fish, and legumes, 37.4%, 28%, and 93.8% of students indicated the correct answer, respectively. Moreover, only 31.3% and 23.4% were aware of the recommendations for the daily consumption of pasta and bread. Furthermore, 40.4% knew that eggs should be eaten twice a week.

When adolescents were asked to rate themselves on their knowledge about dietary habits, measured on a 10-point Likert-type scale, the mean score was 7.0. Almost all the sample agreed that “a balanced diet improves the perceived health status” (97.1%) and “choosing fruit and vegetables supports good health” (98.2%). Almost two-thirds (60.6%) believed that snacks prevent excessive hunger before the next meal, and more than half of the sample (55.7%) believed that having breakfast helps with concentration during the day.

When students were asked about their weekly breakfast habits, 58.5% reported having breakfast during school days. The difference in breakfast habits between boys and girls was statistically significant (*p* = <0.001), with boys being 1.51 times more likely to have breakfast compared to girls (OR = 1.51; 95% CI = 1.2–1.9). Overall, only 32.7% and 22.7% of adolescents consumed fruit and vegetables at least once a day, respectively. Approximately two-thirds of respondents (69.3%) consumed legumes at least twice a week, while only 9.4% had the unhealthy habit of consuming carbonated sugary drinks at least once a day. The dietary indicators used for calculating diet quality scores are summarized in Table 2. Moreover, 59.3% of adolescents had an adequate daily water intake, while 66.6% never/rarely/sometimes added salt to their food, with higher odds among those who had a good diet quality for water intake (OR = 1.55, 95% CI = 1.34–1.78) and added salt (OR = 1.40, 95% CI = 2.23–1.59) (Supplementary File).

When asked which meals adolescents have with at least one parent every day, 17.7% of respondents reported having breakfast, and 43.3% reported having lunch and dinner with them. Moreover, a minority of adolescents reported receiving information on dietary habits from physicians (30.4%). More frequently, adolescents received information on nutrition from parents (55.7%), school (36.8%), and Internet or social media (36.6%). Furthermore, 63.6% expressed an interest in receiving additional information on proper dietary habits.

A multivariate logistic regression model was conducted to identify factors associated with correct dietary habits. The multivariate analysis showed that those who were older than 13 years (OR = 1.73; 95% CI = 1.21–2.47), those who were males (OR = 1.36; 95% CI = 1.02–1.81), those who had breakfast everyday with at least one parent (OR = 2.00; 95% CI = 1.37–2.93), those with higher self-rating dietary habits knowledge (OR = 1.24; 95% CI = 1.13–1.36), those who had knowledge about daily fruit and vegetables portions recommendations (OR = 1.73; 95% CI = 1.27–2.35), those who had correct dietary attitudes (OR = 1.66; 95% CI = 1.23–2.23), those who need to receive additional information about dietary habits (OR = 1.44; 95% CI = 1.08–1.92), those who met the WHO PA recommendations (OR = 1.40; 95% CI = 1.04–1.89), those who spent less than two hours a day watching TV or playing video games (OR = 1.69; 95% CI = 1.26–2.26) were more likely to have a high quality diet score. Additionally, those who reported living with a single family member (OR = 0.34; 95% CI = 0.14–0.81) and those who were current smokers (OR = 0.47; 95% CI = 0.31–0.73) compared with those who had never smoked were negatively associated with having a high quality diet (Table 3).

### 3.3. Adolescents’ Physical Activity

Table 4 provides a detailed summary of the PA assessment, sedentary time, and self-perception of body image. Few respondents (7.4%) were inactive, while more than one-quarter (26.5%) were sufficiently active. Almost two-thirds of the adolescents (66.1%) were considered active or very active, of which more than half were boys (57.4%) and older than thirteen (62.16%). On the other hand, when asked about their sitting time, adolescents spent, on average, 348 min a day sitting. Additionally, 71.4% of respondents met the WHO recommendations on PA.

When participants were asked about their daily habits, 615 (44.4%) spent more than two hours a day watching TV or playing video games, and the vast majority (81.8%) did not follow the WHO recommendations on mobile phone daily time use (less than two hours). A high level of perceived utility of sport was observed, measured on a 10-point Likert-type scale, with a mean value of 9.2. Overall, 1285 (89.7%) participants had received information about PA, of which almost half (49%) from parents, followed by school (37.5%), Internet or social media (37.1%), and physicians (24.5%). Furthermore, more than half (51.6%) expressed the need to receive additional information about PA. When body-image perception was investigated, 716 adolescents (50.4%) were satisfied, slightly more than half were older than thirteen (58.3%; *p* = 0.038), 81.3% had a healthy weight (*p* = 0.001), and more than half (55%) had at least a parent with a university degree (*p* = 0.015). Additionally, 20.3% of the respondents desired to gain weight, while 29.4% preferred to lose weight.

A multivariate logistic regression model was built to identify factors associated with meeting WHO PA recommendations among adolescents. The results showed that adolescents who were older than 13 years (OR = 2.14; 95% CI = 1.54–2.99), who were males (OR = 1.92; 95% CI = 1.46–2.52), who were at risk of alcohol abuse (OR = 1.85; 95% CI = 1.31–2.61), who spent lower hours per day using their mobile phones (OR = 0.93; 95% CI = 0.88–0.99), and who had high quality diet score (OR = 1.35; 95% CI = 1.03–1.78) were more likely to meet the WHO PA recommendations (Table 5).

## 4. Discussion

The current survey, which is part of a large project regarding healthy behaviors among groups of individuals living in Italy, examined knowledge, attitudes, and behaviors on nutrition and physical activities among adolescents, adding inspiring and useful evidence on these topics.

### 4.1. Obesity

First, it is interesting to observe that 18.9% of adolescents in our population are overweight or obese, and these data are lower than those reported in similar studies exploring the same issue in Europe (24.3%) [46] and worldwide (22.2%) [47]. It needs to be highlighted because it is known that obesity in adolescence influences the development of numerous diseases in adulthood [48,49]. It does not appear unnecessary to underline that the Campania region, where this study was conducted, represents one of the regions with the highest percentage of adolescents with obesity in the latest national reports [50]. Over the years, many public health campaigns and programs have been carried out to encourage families in the Campania region to adopt healthier lifestyles and improve children’s nutrition. The presented result seems to be encouraging in describing that these campaigns are probably succeeding. Therefore, there is an increasing need to improve multiprofessional health educational interventions focused on parents and adolescents to enhance adolescents’ nutrition and reduce the risk of obesity-related diseases in adulthood. Exploring their knowledge and eating behaviors, it is clear that adolescents often do not know what healthy foods are, the correct portions, and the need to differentiate the foods during the week. It is interesting to note that only 30.4% report having received information about their eating habits from a physician. Previous literature showed that adolescents and adults who receive information about nutrition from healthcare professionals are more likely to follow proper diets [51]. Since the assumption of healthy eating habits is important in adolescence because it leads to correct behaviors in adulthood [52], the school can play a fundamental role since numerous studies have described that following a correct diet at school helps to reduce body weight and improve, therefore, knowledge and long-term correct behaviors [53].

### 4.2. Smoking and Alcohol Use

Second, it is certainly worrying to note that one-third of the adolescents surveyed have smoked or are smokers and are at risk of alcohol abuse. These data are similar to those reported in similar surveys of other European or international populations [54,55]. The presented adolescent generation, compared to previous ones, seems to have a greater awareness of the risks of smoking, probably thanks to information campaigns that have been carried out in recent years on media channels easily accessible to young people, but it is not sufficient to eradicate smoking among adolescents [56]. Moreover, the high percentage of adolescents at risk of alcohol abuse is alarming. The international scientific community is putting emphasis on this concerning trend in adolescents, and several international studies have already pointed out that the percentage of adolescents and pre-adolescents who approach alcohol in dangerous doses is increasing [57,58]. Alcohol use in youth predisposes to numerous health problems in adulthood [59]. It is necessary to promote public health strategies to inform adolescents and work to reduce this spreading trend.

### 4.3. Physical Activity and Dietary Habits

Third, exploring PA, 66.1% of adolescents had an active or very active IPAQ-SF score. The high percentage of active adolescents may have influenced the high level of perceived utility of sport observed (mean value of 9.2). Boys and those older than thirteen are more active. Although data regarding adolescents who did not meet WHO PA recommendations (28.6%) seem to be encouraging because they are lower than those reported in other European regions (66.6%) [60] or globally (around 80%) [61], they had sedentary behaviors. Indeed, adolescents spent a lot of time sitting, did not follow WHO recommendations for mobile phone daily time use, and spent more than two hours a day watching TV or playing video games. WHO has described that, globally, 80% of adolescents do not meet the recommended levels of PA [62]. Results from the Global Matrix 3.0, including 49 countries, found that PA levels of children and adolescents are low and screen time levels are high, with only 27–33% and 34–39% meeting the PA and screen time recommendations, respectively [63]. The risks of a sedentary lifestyle and future health problems should be considered since higher screen time in adolescence has been associated with cardiometabolic disease in adulthood [64], reduced quality of life [65], and difficulties in social activities [66]. It has been shown that, especially after the COVID-19 pandemic, there are increasing percentages of adolescents who spend more time with electronic devices and have difficulties socializing with peers. Indeed, school closures and restricted access to community services and activities for adolescents caused a spread in screen time behaviors during the COVID-19 pandemic. Evidence indicates that rates of PA in adolescents declined during the early stages of the pandemic while recreational screen time and sedentary behaviors increased [67,68,69]. Moreover, the role of health professionals in informing adolescents about the importance of PA is insufficient; indeed, only 24.5% declared to have been informed on this topic by a physician. Therefore, it is clear that public health interventions are needed to encourage communication on this topic between adolescents and healthcare workers. Another aspect not to be underestimated is the adolescents’ perception of their body image; indeed, about half of them are not satisfied and eager to put on or lose weight. It has been shown how the excessive images that are transmitted through the mass media have caused, in recent years, the creation of ideals of bodily perfection, which is hardly achievable by adolescents, and they are constantly feeling inadequate to the standards imposed by society [70,71].

Fourth, it appears useful to discuss the results of multivariate regression models. The data presented described that those who were older than 13 years and were males were more likely to have high quality diet scores and to meet recommendations on PA. This can be explained by the fact that older adolescents are more aware of quality diets and the need to perform physical activities. Moreover, previous literature confirms that male adolescents are more active, consume significantly more fruit and vegetables than female adolescents, and that females have a much higher chance of negative emotional eating [60,72,73]. It is interesting to observe that those who had breakfast every day with at least one parent, those who rated themselves highly on their healthy dietary habits knowledge, those who knew daily fruit and vegetables portion recommendations, and those who had correct dietary attitudes were more likely to have high quality diet scores. It has been described that it is important that parents are more present and have more knowledge and positive attitudes on dietary behaviors. In this way, parents positively impact their adolescents’ eating habits regarding home-prepared meals and the availability of fresh ingredients to cook food at home [74,75,76,77]. Moreover, our findings confirm that meeting WHO PA recommendations and having high quality diet scores are correlated and should be clinically meaningful; indeed, it is well known that adolescents who had experienced positive health outcomes in terms of physical fitness because of good dietary habits were more likely to perceive the importance of healthy eating [77]. Furthermore, those who spend less than two hours a day watching TV or playing video games are more likely to have high quality diet scores, confirming other studies that described how excessive screen time viewing is correlated with increased risk for obesity, unhealthy dietary habits, eating disorders, and less physical exercise [78]. Furthermore, those who need to receive additional information about dietary habits are more likely to have high quality diet scores, and this is in line with studies that described how food literacy allows adolescents to use the information to make healthy and sustainable food-related decisions [52]. Moreover, those who are current smokers are more likely to have low-quality diet scores. This is confirmed by previous literature describing that unhealthy behaviors, such as alcohol, drug, and tobacco use, are associated with a worse diet intake [79]. Lastly, those who reported living with a single family member are more likely to have low-quality diet scores, confirming literature describing that adolescents living in two-parent families report better diet behaviors than those in single-parent ones [80]. In our results, those who were at risk of alcohol dependence were more likely to meet WHO’s PA recommendations. This data could appear strange, but, unfortunately, previous literature has described that some sports are associated with alcohol consumption among adolescents [81]. It is important to highlight that there are psychological and social potential factors for adolescents’ poor dietary knowledge. It has been well described the importance of nutrition interventions to make adolescents aware of food intake’s role in decreasing depressive symptoms or preventing further symptom exacerbation [82]. Indeed, adolescents with major depressive disorders are more likely to consume less healthy meals compared with healthy children [83]. Moreover, different social factors related to family and peers could have significant impacts on adolescent food knowledge and, consequently, choices: parental control, family’s food habits, peer pressure due to socialization, busy school schedules, and conformity [84].

### 4.4. Limitation

This study presents several limitations expected from the study design. First, since this is a cross-sectional study, it is possible only to observe associations between the determinants and the different outcomes of interest, but not attribution of cause and effect. Second, regarding attitudes, respondents may have been influenced to give the most “desirable” answers, producing an overestimation of the positive attitudes and behaviors toward healthy practices. For example, almost the entire sample agrees that a balanced diet improves health. Regarding the reporting of sensitive behaviors, such as smoking and alcohol consumption, adolescents can underreport undesirable behaviors and overreport positive attitudes or healthy practices, determining the social desirability bias. To contain these limitations, the questionnaire was self-administered, and, therefore, the confidentiality and anonymity of responses were guaranteed. Third, since adolescents’ behaviors and health-related information were investigated, a recall bias may have occurred for this information. Fourth, the sample may raise issues of generalizability. Indeed, we collected data only in the Campania Region, and our results could not be generalizable to all Italian adolescents. Finally, since the questionnaire was completed at school, adolescents could have been influenced by their friends. Despite these limitations, a high response rate was achieved, and the findings of this study provide important insights and original knowledge on a public health issue that has a relevant impact not only on adolescents’ health but also on future adults.

## 5. Conclusions

Projects aiming to promote healthy behaviors can empower adolescents to acquire healthy eating habits, perform physical activities, and stop smoking or consuming alcohol. In this way, adolescents can sustain a healthy lifestyle into adulthood. Notably, the majority of adolescents need to improve their knowledge regarding the intake of all recommended foods and the importance of an active life. The present results highlight the need for practical implications regarding public health interventions. Schools are places where adolescents spend many hours, and teachers have the important role of giving adolescents correct information in synergy with public healthcare programs and families. In particular, schools need to implement courses on healthy diet habits, promote PA programs involving families and teachers, and spread knowledge on the risks associated with the excessive use of electronic devices.

## Figures and Tables

**Table 1:** Adolescents’ socio-demographic and health-related characteristics (*N* = 1433).

Socio-Demographic and Health-Related Characteristics	Total		Diet Quality				WHO PA Recommendations			
			Poor (*n*: 610, 44.1%)		Good (*n*: 774, 55.9%)		Not Meeting (*n*: 375, 28.6%)		Meeting (*n*: 938, 71.4%)	
	*N*	%	*N*	%	*N*	%	*N*	%	*N*	%
***Age group*** (1429) *	15.2 ± 2.6 (11–19) **		*t*-test = −0.21, *df* = 1378, *p =* 0.8362				*t*-test = −10.35, *df* = 1309, *p =* <0.001			
≤13 years	557	39	248	46.4	287	53.6	213	40	319	60
>13 years	872	61	361	42.7	484	57.3	162	20.8	617	79.2
			*χ*^2^ = 1.75, *df* = 1, *p* = 0.185				*χ*^2^ = 57.31, *df* = 1, *p* = <0.001			
***Gender*** (1422) *										
Female	704	49.5	311	45.7	369	54.3	232	35.7	417	64.3
Male	718	50.5	294	42.4	399	57.6	142	78.3	512	21.7
			*χ*^2^ = 1.53, *df* = 1, *p* = 0.217				*χ*^2^ = 31.35, *df* = 1, *p* = <0.001			
***Nationality*** (1425) *										
Foreigner	45	3.2	18	42.9	24	57.1	7	16.7	35	83.3
Italian	1380	96.8	586	43.9	748	56.1	367	29	899	71
			*χ*^2^ = 0.02, *df* = 1, *p* = 0.890				*χ*^2^ = 3.02, *df* = 1, *p* = 0.082			
***Family members*** (1421) *										
Single member	48	3.4	12	25.5	35	74.5	9	20	36	80
More than one	1373	96.6	594	44.8	732	55.2	364	28.9	894	71.1
			*χ*^2^ = 6.83, *df* = 1, *p* = 0.009				*χ*^2^ = 1.70, *df* = 1, *p* = 0.193			
***Parents’ educational level***										
Other	689	48.1	313	47.1	351	52.9	176	28.5	442	71.5
At least one with a university degree	744	51.9	297	41.3	423	58.7	199	28.6	496	71.4
			*χ*^2^ = 4.86, *df* = 1, *p* = 0.027				*χ*^2^ = 0.004, *df* = 1, *p* = 0.951			
***Parents’ occupation***										
Other	69	4.8	31	47.7	34	52.3	18	31	40	69
At least one employed	1364	95.2	579	43.9	740	56.1	357	28.5	898	71.5
			*χ*^2^ = 0.36, *df* = 1, *p* = 0.547				*χ*^2^ = 0.18, *df* = 1, *p* = 0.670			
***Weight status*** (1257) *										
Underweight	59	4.7	28	29.1	29	50.9	17	30.9	38	69.1
Healthy weight	972	77.3	409	43.3	536	56.7	238	26.4	663	73.6
Overweight/obese	226	18	93	42.3	127	57.7	66	31.3	145	68.7
			*χ*^2^ = 0.88, *df* = 2, *p* = 0.644				*χ*^2^ = 2.36, *df* = 2, *p* = 0.307			
***NCDs*** (1418) *										
No	1179	83.2	501	43.9	640	56.1	321	29.6	762	70.4
At least one	239	16.8	102	44.5	127	55.5	51	23.2	169	76.8
			*χ*^2^ = 0.03, *df* = 1, *p* = 0.860				*χ*^2^ = 3.74, *df* = 1, *p* = 0.053			
***Medication*** (232) *^a^										
No	108	46.5	51	49.5	52	50.5	21	21	79	79
Yes	124	53.5	49	41.5	69	58.5	29	25.9	83	74.1
			*χ*^2^ = 1.42, *df* = 1, *p* = 0.234				*χ*^2^ = 0.70, *df* = 1, *p* = 0.402			
***Parents’ NCDs*** (1405) *										
No	1112	79.2	487	45.3	588	54.7	298	29.4	715	70.6
At least one	293	20.8	110	39.2	171	60.8	70	25.2	208	74.8
			*χ*^2^ = 3.43, *df* = 1, *p* = 0.064				*χ*^2^ = 1.92, *df* = 1, *p* = 0.166			
***Self-perceived health status*** (1345) *	8.1 ± 1.5 (1–10) **		*t*-test = −4.21, *df* = 1299, *p =* <0.001				*t*-test = 0.46, *df* = 1240, *p =* 0.6491			
Poor	11	0.8	6	54.5	5	45.5	1	11.1	8	88.9
Fair	24	1.8	16	66.7	8	33.3	6	26.1	17	73.9
Good	144	10.7	81	57.9	59	42.1	39	31.2	86	68.8
Very good	584	43.4	231	41.2	329	58.8	137	25.5	400	74.5
Excellent	582	43.3	225	39.7	341	60.3	161	29.4	387	70.6
			*χ*^2^ = 21.83, *df* = 4, *p* = <0.001				*χ*^2^ for trend =0.68, *p* = 0.4111			
***Being at risk of alcohol abuse*** (1379) *										
No	768	55.7	314	42.6	423	57.4	266	37.1	452	62.9
Yes	611	44.3	272	45.7	323	54.3	95	17.2	458	82.8
			*χ*^2^ = 1.29, *df* = 1, *p* = 0.256				*χ*^2^ = 60.64, *df* = 1, *p* = <0.001			
***Smoking habits*** (1406) *										
Never	915	65.1	366	41.5	516	58.5	287	33.3	574	66.7
Former	272	19.4	119	44.4	149	55.6	56	22.3	195	77.7
Current	219	15.5	112	53.3	98	46.7	26	14.4	155	85.6
			*χ*^2^ = 9.68, *df* = 2, *p* = 0.008				*χ*^2^ = 32.3097, *df* = 2, *p* = <0.001			

* In brackets are the number of respondents to each item. ** Mean ± Standard Deviation (Range). ^a^ Only among those who had NCDs.

**Table 2:** Dietary indicators for the diet quality score (*N* = 1433).

Diet Quality Score	*N*	%
**Breakfast during school days** (1425) *		
No	592	41.5
Yes	833	58.5
**Fruit at least once a day** (1427) *		
No	961	67.3
Yes	466	32.7
**Vegetables at least once a day** (1417) *		
No	1095	77.3
Yes	322	22.7
**Legumes at least twice a week** (1409) *		
No	432	30.7
Yes	977	69.3
**Carbonated sugary drinks less than once a day** (1426) *		
No	134	9.4
Yes	1292	90.6
**Number of correct dietary habits** (1384) *		
0	19	1.4
1	160	11.6
2	431	31.1
3	420	30.4
4	258	18.6
5	96	6.9
**Correct dietary habits** (1384) *		
Less than 3	610	44.1
At least 3	774	55.9

* In brackets are the number of respondents to each item.

**Table 3:** Multivariate logistic regression model about adolescents who were more likely to have high quality diet scores (Model 1).

Variables	OR	95% CI	*p*
Model 1. Adolescents who were more likely to have high quality diet scores. Log likelihood = −644.90, *χ* ^2^ = 126.61 (15 *df*), *p =* < 0.001, No. of obs = 1047			
Older than 13 years	1.73	1.21–2.47	0.003
Living with a single family member	0.34	0.14–0.81	0.015
Males	1.36	1.02–1.81	0.036
At least one parent with a university degree	1.15	0.88–1.51	0.316
Never smoker	1 *		
Current smokers	0.47	0.31–0.73	0.001
Former smokers	0.73	0.50–1.06	0.095
Breakfast with at least one parent	2.00	1.37–2.93	<0.001
Higher self-rating of healthy dietary habits knowledge	1.24	1.13–1.36	<0.001
Correct knowledge about daily fruit and vegetables portions	1.73	1.27–2.35	0.001
Correct dietary attitudes	1.66	1.23–2.23	0.001
Need to receive additional information about dietary habits	1.44	1.08–1.92	0.014
At least one source of dietary information	1.74	0.96–3.18	0.070
Meeting WHO PA recommendations	1.40	1.04–1.89	0.026
<2 h per day watching TV/playing video games	1.69	1.26–2.26	<0.001
<2 h per day using mobile phone	1.32	0.90–1.94	0.148

OR = Odds Ratio; CI = confidence interval. * Reference category. The following variable was deleted by the backward elimination procedure: lunch and dinner with at least one parent.

**Table 4:** Adolescents’ physical activity assessment, sedentary time, and self-perception of body image (*N* = 1433).

Physical Activity and Body Image Score	*N*	%
***IPAQ score*** (1123) *		
Inactive	83	7.4
Sufficiently active	298	26.5
Active/very active	742	66.1
***Sitting time (min)*** (1055) *	348.4 ± 215.1 (60–960) **	
***WHO recommendations on PA*** (1313) *		
No	375	28.6
Yes	938	71.4
***WHO recommendations on screen time usage*** (1385) *		
No (≥2 h/day)	615	44.4
Yes (<2 h/day)	770	55.6
***WHO recommendations on mobile phone time usage*** (1386) *		
No (≥2 h/day)	1134	81.8
Yes (<2 h/day)	252	18.2
***Perceived utility of sport*** (998) *	9.2 ± 1.2 (0–10) **	
***Self-perception of body image*** (1422) *		
Satisfied	716	50.4
Unsatisfied	706	49.6

* In brackets are the number of respondents to each item. ** Mean ± Standard Deviation (Range).

**Table 5:** Multivariate logistic regression model about adolescents who meet WHO physical activity recommendations (Model 2).

Variables	OR	95% CI	*p*
Model 2. Adolescents who meet WHO physical activity recommendations Log likelihood = −632.36, *χ*^2^ = 115.84 (8 *df*), *p =* < 0.001, No. of obs = 1164			
Older than 13 years old	2.14	1.54–2.99	<0.001
Males	1.92	1.46–2.52	<0.001
At least one NCD	1.40	0.96–2.05	0.081
Risk of alcohol abuse	1.85	1.31–2.61	<0.001
Never smoker	1 *		
Current smokers	1.60	0.95–2.70	0.078
High quality diet score	1.35	1.03–1.78	0.031
Lower hours spent per day using mobile phones	0.93	0.88–0.99	0.017
At least one source of PA information	0.65	0.39–1.08	0.102

OR = Odds Ratio; CI = confidence interval. * Reference category. The following variable was deleted by the backward elimination procedure: living with single family members and former smokers.

## Data Availability

The data presented in this study are available upon request from the corresponding author due to ethical restrictions.

## Supplementary Materials

The following supporting information can be downloaded at: https://www.mdpi.com/article/10.3390/nu16234162/s1, Figure S1: Distribution of correct dietary habits by sex: score from 0 to 5.

## References

[1] GBD 2019 Diseases and Injuries Collaborators (2020). Global burden of 369 diseases and injuries in 204 countries and territories, 1990-2019: A systematic analysis for the Global Burden of Disease Study 2019. Lancet.

[2] Istituto Superiore di Sanità Chronic Diseases and Aging. https://www.iss.it/web/iss-en/chronic-diseases-and-aging1.

[3] Ministero della Salute Direzione Generale della Prevenzione Sanitaria. Piano Nazionale della Prevenzione 2020–2025. https://www.salute.gov.it/imgs/C_17_pubblicazioni_2955_allegato.pdf.

[4] Di Cesare M., Sorić M., Bovet P., Miranda J.J., Bhutta Z., Stevens G.A., Laxmaiah A., Kengne A.P., Bentham J. (2019). The epidemiological burden of obesity in childhood: A worldwide epidemic requiring urgent action. BMC Med..

[5] van Sluijs E.M.F., Ekelund U., Crochemore-Silva I., Guthold R., Ha A., Lubans D., Oyeyemi A.L., Ding D., Katzmarzyk P.T. (2021). Physical activity behaviours in adolescence: Current evidence and opportunities for intervention. Lancet.

[6] Seo Y.G., Lim H., Kim Y., Ju Y.S., Lee H.J., Jang H.B., Park S.I., Park K.H. (2019). The effect of a multidisciplinary lifestyle intervention on obesity status, body composition, physical fitness, and cardiometabolic risk markers in children and adolescents with obesity. Nutrients.

[7] Lobstein T., Jackson-Leach R., Moodie M.L., Hall K.D., Gortmaker S.L., Swinburn B.A., James W.P., Wang Y., McPherson K. (2015). Child and adolescent obesity: Part of a bigger picture. Lancet.

[8] Juonala M., Magnussen C.G., Berenson G.S., Venn A., Burns T.L., Sabin M.A., Srinivasan S.R., Daniels S.R., Davis P.H., Chen W. (2011). Childhood adiposity, adult adiposity, and cardiovascular risk factors. N. Engl. J. Med..

[9] Litwin S.E. (2014). Childhood obesity and adulthood cardiovascular disease: Quantifying the lifetime cumulative burden of cardiovascular risk factors. J. Am. Coll. Cardiol..

[10] Lai C.C., Sun D., Cen R., Wang J., Li S., Fernandez-Alonso C., Chen W., Srinivasan S.R., Berenson G.S. (2014). Impact of long-term burden of excessive adiposity and elevated blood pressure from childhood on adulthood left ventricular remodeling patterns: The Bogalusa Heart Study. J. Am. Coll. Cardiol..

[11] Lee I.M., Shiroma E.J., Lobelo F., Puska P., Blair S.N., Katzmarzyk P.T., Lancet Physical Activity Series Working Group (2012). Effect of physical inactivity on major non-communicable diseases worldwide: An analysis of burden of disease and life expectancy. Lancet.

[12] Pizzi M.A., Vroman K. (2013). Childhood obesity: Effects on children’s participation, mental health, and psychosocial development. Occup. Ther. Health Care.

[13] Popkin B.M., Hawkes C. (2016). Sweetening of the global diet, particularly beverages: Patterns, trends, and policy responses. Lancet Diabetes Endocrinol..

[14] Ebbeling C.B., Feldman H.A., Chomitz V.R., Antonelli T.A., Gortmaker S.L., Osganian S.K., Ludwig D.S. (2012). A randomized trial of sugar-sweetened beverages and adolescent body weight. N. Engl. J. Med..

[15] Pileggi C., Lotito F., Bianco A., Nobile C.G., Pavia M. (2013). Relationship between chronic short sleep duration and childhood Body Mass Index: A school-based cross-sectional study. PLoS ONE.

[16] Pileggi C., Carbone V., Nobile C.G., Pavia M. (2015). Blood pressure and related cardiovascular disease risk factors in 6-18 year-old students in Italy. J. Paediatr. Child. Health.

[17] Pelullo C.P., Di Giuseppe G. (2018). Vaccinations among Italian adolescents: Knowledge, attitude and behavior. Hum. Vaccin. Immunother..

[18] Di Giuseppe G., Pelullo C.P., Mitidieri M., Lioi G., Pavia M. (2020). Cancer prevention: Knowledge, attitudes and lifestyle cancer-related behaviors among adolescents in Italy. Int. J. Environ. Res. Public Health.

[19] Salam R.A., Padhani Z.A., Das J.K., Shaikh A.Y., Hoodbhoy Z., Jeelani S.M., Lassi Z.S., Bhutta Z.A. (2020). Effects of lifestyle modification interventions to prevent and manage child and adolescent obesity: A systematic review and meta-analysis. Nutrients.

[20] Hamulka J., Wadolowska L., Hoffmann M., Kowalkowska J., Gutkowska K. (2018). Effect of an education program on nutrition knowledge, attitudes toward nutrition, diet quality, lifestyle, and body composition in polish teenagers. The ABC of Healthy Eating Project: Design, protocol, and methodology. Nutrients.

[21] Meng Y., Manore M.M., Schuna J.M., Patton-Lopez M.M., Branscum A., Wong S.S. (2018). Promoting healthy diet, physical activity, and life-skills in high school athletes: Results from the wave ripples for change childhood obesity prevention two-year intervention. Nutrients.

[22] Chaudhary A., Sudzina F., Mikkelsen B.E. (2020). Promoting healthy eating among young people-a review of the evidence of the impact of school-based interventions. Nutrients.

[23] Jemmott J.B., Zhang J., Jemmott L.S., Icard L.D., Ngwane Z., Makiwane M., O’Leary A. (2019). Intervention increases physical activity and healthful diet among south african adolescents over 54 months: A randomized controlled trial. J. Adolesc. Health.

[24] Pandit-Agrawal D., Khadilkar A., Chiplonkar S., Khadilkar V. (2018). Knowledge of nutrition and physical activity in apparently healthy Indian adults. Public Health Nutr..

[25] Ranasinghe P., Pigera A.S., Ishara M.H., Jayasekara L.M., Jayawardena R., Katulanda P. (2015). Knowledge and perceptions about diet and physical activity among Sri Lankan adults with diabetes mellitus: A qualitative study. BMC Public Health.

[26] Lamore K., Ducrot P., Latino-Martel P., Soler M., Foucaud J. (2019). Diet, physical activity, obesity, and breastfeeding: How french people perceive factors associated with cancer risk. Nutrients.

[27] Florindo A.A., Brownson R.C., Mielke G.I., Gomes G.A., Parra D.C., Siqueira F.V., Lobelo F., Simoes E.J., Ramos L.R., Bracco M.M. (2015). Association of knowledge, preventive counseling and personal health behaviors on physical activity and consumption of fruits or vegetables in community health workers. BMC Public Health.

[28] Partida S., Marshall A., Henry R., Townsend J., Toy A. (2018). Attitudes toward nutrition and dietary habits and effectiveness of nutrition education in active adolescents in a private school setting: A pilot study. Nutrients.

[29] Rawal T., Willeboordse M., Arora M., Sharma N., Nazar G.P., Tandon N., van Schayck C.P. (2021). Prevalence of excessive weight and underweight and its associated knowledge and lifestyle behaviors among urban private school-going adolescents in New Delhi. Nutrients.

[30] Gamage A.U., Jayawardana P.L. (2017). Knowledge of non-communicable diseases and practices related to healthy lifestyles among adolescents, in state schools of a selected educational division in Sri Lanka. BMC Public Health.

[31] Aday L.A., Cornelius L.J. (2000). Designing and conducting health surveys: A comprehensive guide.

[32] Cacciari E., Milani S., Balsamo A., Spada E., Bona G., Cavallo L., Cerutti F., Gargantini L., Greggio N., Tonini G. (2006). Italian cross-sectional growth charts for height, weight and BMI (2 to 20 yr). J. Endocrinol. Investig..

[33] Vereecken C.A., Maes L. (2003). A Belgian study on the reliability and relative validity of the Health Behaviour in School-Aged Children food-frequency questionnaire. Public Health Nutr..

[34] Roberts C., Freeman J., Samdal O., Schnohr C.W., de Looze M.E., Nic Gabhainn S., Iannotti R., Rasmussen M., International HBSC Study Group (2009). The Health Behaviour in School-aged Children (HBSC) study: Methodological developments and current tensions. Int. J. Public Health.

[35] World Health Organization Young People’s Health in Context. Health Behaviour in School-Aged Children (HBSC) Study: International Report from the 2001/2002 Survey. https://iris.who.int/bitstream/handle/10665/107560/e82923.pdf?sequence=1.

[36] Nardone P., Pierannunzio D., Ciardullo S., Lazzeri G., Cappello N., Spinelli A., the 2018 HBSC-Italia Group (2020). Dietary habits among Italian adolescents and their relation to socio-demographic characteristics. Ann. Ist. Super. Sanita.

[37] Lee P.H., Macfarlane D.J., Lam T.H., Stewart S.M. (2011). Validity of the International Physical Activity Questionnaire Short Form (IPAQ-SF): A systematic review. Int. J. Behav. Nutr. Phys. Act..

[38] Sjostrom M., Ainsworth B.E., Bauman A., Bull F.C., Hamilton-Craig C.R., Sallis J.F. Guidelines for data processing analysis of the International Physical Activity Questionnaire (IPAQ)-Short and Long Forms. https://www.researchgate.net/file.PostFileLoader.html?id=5641f4c36143250eac8b45b7&assetKey=AS%3A294237418606593%401447163075131.

[39] World Health Organization WHO guidelines on physical activity and sedentary behaviour. https://iris.who.int/bitstream/handle/10665/336656/9789240015128-eng.pdf?sequence=1.

[40] Lear S.A., Hu W., Rangarajan S., Gasevic D., Leong D., Iqbal R., Casanova A., Swaminathan S., Anjana R.M., Kumar R. (2017). The effect of physical activity on mortality and cardiovascular disease in 130 000 people from 17 high-income, middle-income, and low-income countries: The PURE study. Lancet.

[41] European Commission (2021). Recommendations on Sedentary Time for Children and Adolescents. https://knowledge4policy.ec.europa.eu/health-promotion-knowledge-gateway/physical-activity-sedentary-behaviour-table-3a_en.

[42] Hoelscher D.M., Day R.S., Kelder S.H., Ward J.L. (2003). Reproducibility and validity of the secondary level School-Based Nutrition Monitoring student questionnaire. J. Am. Diet. Assoc..

[43] Agenzia Nazionale per i Servizi Sanitari Regionali (AGENAS) La promozione Della SaluteNel Terzo Millennio: Facebook, Social Gaming ePromozione di Stili di Vita Sani tra Gli Adolescenti. I Questionari Per la Sorveglianza Nutrizionale Negli Adolescenti. https://sinu.it/wp-content/uploads/2020/02/I-QUESTIONARI-PER-LA-SORVEGLIANZA-NUTRIZIONALE-DEGLI-ADOLESCENTI.pdf.

[44] Stata Corporation (2017). Stata Reference Manual Release 17.

[45] Hosmer D.W., Lemeshow S. (2000). Applied Logistic Regression.

[46] Kontochristopoulou A.M., Karatzi K., Karaglani E., Cardon G., Kivelä J., Iotova V., Tankova T., Rurik I., Radone A.S., Liatis S. (2024). Parental practices and children’s lifestyle correlates of childhood overweight/obesity in Europe: The Feel4Diabetes study. J. Hum. Nutr. Diet..

[47] Vazquez C.E., Cubbin C. (2020). Socioeconomic status and childhood obesity: A review of literature from the past decade to inform intervention research. Curr. Obes. Rep..

[48] Horesh A., Tsur A.M., Bardugo A., Twig G. (2021). Adolescent and childhood obesity and excess morbidity and mortality in young adulthood-a systematic review. Curr. Obes. Rep..

[49] Norris S.A., Frongillo E.A., Black M.M., Dong Y., Fall C., Lampl M., Liese A.D., Naguib M., Prentice A., Rochat T. (2022). Nutrition in adolescent growth and development. Lancet.

[50] Epicentro Istituto Superiore di Sanità. OKkio Alla SALUTE: I Risultati Dell’Indagine 2023 in Campania. https://www.epicentro.iss.it/okkioallasalute/report-regionale-2023/campania-2023.pdf.

[51] Hargreaves D., Mates E., Menon P., Alderman H., Devakumar D., Fawzi W., Greenfield G., Hammoudeh W., He S., Lahiri A. (2022). Strategies and interventions for healthy adolescent growth, nutrition, and development. Lancet.

[52] Ares G., De Rosso S., Mueller C., Philippe K., Pickard A., Nicklaus S., van Kleef E., Varela P. (2024). Development of food literacy in children and adolescents: Implications for the design of strategies to promote healthier and more sustainable diets. Nutr. Rev..

[53] Diamantis D.V., Linos A., Hu F.B., Veloudaki A., Petralias A., Leung C.W. (2024). Impact of a school-based food assistance program on household food insecurity in Greece, 2012–2019: A multi-year evaluation of the DIATROFI program. Lancet Reg. Health Eur..

[54] Vashishtha R., Pennay A., Dietze P., Marzan M.B., Room R., Livingston M. (2021). Trends in adolescent drinking across 39 high-income countries: Exploring the timing and magnitude of decline. Eur. J. Public Health.

[55] Ma C., Xi B., Li Z., Wu H., Zhao M., Liang Y., Bovet P. (2021). Prevalence and trends in tobacco use among adolescents aged 13–15 years in 143 countries, 1999-2018: Findings from the Global Youth Tobacco Surveys. Lancet Child Adolesc. Health.

[56] Meza R., Jimenez-Mendoza E., Levy D.T. (2020). Trends in tobacco use among adolescents by grade, sex, and race, 1991-2019. JAMA Netw. Open.

[57] World Health Organization Alcohol. https://www.who.int/health-topics/alcohol.

[58] World Health Organization Alcohol, e-Cigarettes, Cannabis: Concerning Trends in Adolescent Substance Use, Shows New WHO/Europe Report. https://www.who.int/europe/news/item/25-04-2024-alcohol--e-cigarettes--cannabis--concerning-trends-in-adolescent-substance-use--shows-new-who-europe-report.

[59] Boden J., Blair S., Newton-Howes G. (2020). Alcohol use in adolescents and adult psychopathology and social outcomes: Findings from a 35-year cohort study. Aust. N. Z. J. Psychiatry.

[60] Romero-Parra N., Solera-Alfonso A., Bores-García D., Delfa-de-la-Morena J.M. (2023). Sex and educational level differences in physical activity and motivations to exercise among Spanish children and adolescents. Eur. J. Pediatr..

[61] Guthold R., Stevens G.A., Riley L.M., Bull F.C. (2020). Global trends in insufficient physical activity among adolescents: A pooled analysis of 298 population-based surveys with 1.6 million participants. Lancet Child Adolesc. Health.

[62] World Health Organization Physical Activity. https://who.int/news-room/fact-sheets/detail/physical-activity.

[63] Aubert S., Barnes J.D., Abdeta C., Abi Nader P., Adeniyi A.F., Aguilar-Farias N., Andrade Tenesaca D.S., Bhawra J., Brazo-Sayavera J., Cardon G. (2018). Global matrix 3.0 physical activity report card grades for children and youth: Results and analysis from 49 countries. J. Phys. Act. Health.

[64] Nagata J.M., Lee C.M., Lin F., Ganson K.T., Pettee Gabriel K., Testa A., Jackson D.B., Dooley E.E., Gooding H.C., Vittinghoff E. (2023). Screen time from adolescence to adulthood and cardiometabolic disease: A prospective cohort study. J. Gen. Intern. Med..

[65] Lavados-Romo P., Andrade-Mayorga O., Morales G., Muñoz S., Balboa-Castillo T. (2023). Association of screen time and physical activity with health-related quality of life in college students. J. Am. Coll. Health.

[66] Twenge J.M., Campbell W.K. (2018). Associations between screen time and lower psychological well-being among children and adolescents: Evidence from a population-based study. Prev. Med. Rep..

[67] Santos R.M.S., Mendes C.G., Sen Bressani G.Y., de Alcantara Ventura S., de Almeida Nogueira Y.J., de Miranda D.M., Romano-Silva M.A. (2023). The associations between screen time and mental health in adolescents: A systematic review. BMC Psychol..

[68] Choi E.J., King G.K.C., Duerden E.G. (2023). Screen time in children and youth during the pandemic: A systematic review and meta-analysis. Glob. Pediatr..

[69] Lua V.Y.Q., Chua T.B.K., Chia M.Y.H. (2023). A narrative review of screen time and wellbeing among adolescents before and during the covid-19 pandemic: Implications for the future. Sports.

[70] Di Gesto C., Matera C., Policardo G.R., Nerini A. (2023). Instagram as a digital mirror: The effects of instagram likes and disclaimer labels on self-awareness, body dissatisfaction, and social physique anxiety among young Italian women. Curr. Psychol..

[71] Mohsenpour M.A., Karamizadeh M., Barati-Boldaji R., Ferns G.A., Akbarzadeh M. (2023). Structural equation modeling of direct and indirect associations of social media addiction with eating behavior in adolescents and young adults. Sci. Rep..

[72] Saat N.Z.M., Hanawi S.A., Chew N.H.H., Ahmad M., Farah N.M.F., Kadar M., Yahya H.M., Warif N.M.A., Daud M.K.M. (2023). The association of eating behaviour with physical activity and screen time among adolescents in the Klang Valley, Malaysia: A cross-sectional study. Healthcare.

[73] Sze K.Y., Lee E.K., Chan R.H., Kim J.H. (2021). Prevalence of negative emotional eating and its associated psychosocial factors among urban Chinese undergraduates in Hong Kong: A cross-sectional study. BMC Public Health.

[74] Liu K.S.N., Chen J.Y., Sun K.-S., Tsang J.P.Y., Ip P., Lam C.L.K. (2023). Family facilitators of barriers to and strategies for healthy eating among Chinese adolescents: Qualitative interviews with parent–adolescent dyads. Nutrients.

[75] Yee A.Z., Lwin M.O., Ho S.S. (2017). The influence of parental practices on child promotive and preventive food consumption behaviors: A systematic review and meta-analysis. Int. J. Behav. Nutr. Phys. Act..

[76] Liu K.S.N., Chen J.Y., Ng M.Y.C., Yeung M.H.Y., Bedford L.E., Lam C.L.K. (2021). How does the family influence adolescent eating habits in terms of knowledge, attitudes and practices? A global systematic review of qualitative studies. Nutrients.

[77] Liu K.S.N., Chen J.Y., Sun K.S., Tsang J.P.Y., Ip P., Lam C.L.K. (2022). Adolescent knowledge, attitudes and practices of healthy eating: Findings of qualitative interviews among Hong Kong families. Nutrients.

[78] Priftis N., Panagiotakos D. (2023). Screen time and its health consequences in children and adolescents. Children.

[79] Mesas A.E., Girotto E., Rodrigues R., Martínez-Vizcaíno V., Jiménez-López E., López-Gil J.F. (2023). Ultra-processed food consumption is associated with alcoholic beverage drinking, tobacco smoking, and illicit drug use in adolescents: A nationwide population-based study. Int. J. Ment. Health Addiction.

[80] Lazzeri G., Ciardullo S., Spinelli A., Pierannunzio D., Dzielska A., Kelly C., Thorsteinsson E.B., Qirjako G., Geraets A., Ojala K. (2023). The correlation between adolescent daily breakfast consumption and socio-demographic: Trends in 23 European countries participating in the Health Behaviour in School-Aged Children Study (2002–2018). Nutrients.

[81] Moore M.J., Werch C.E. (2005). Sport and physical activity participation and substance use among adolescents. J. Adolesc. Health.

[82] Wang Y., Liu J., Compher C., Kral T.V.E. (2022). Associations between dietary intake, diet quality and depressive symptoms in youth: A systematic review of observational studies. Health Promot. Perspect..

[83] Korczak D.J., Perruzza S., Chandrapalan M., Cost K., Cleverley K., Birken C.S., McCrindle B.M. (2022). The association of diet and depression: An analysis of dietary measures in depressed, non-depressed, and healthy youth. Nutr. Neurosci..

[84] Louey J., He J., Partridge S.R., Allman-Farinelli M. (2024). Facilitators and barriers to healthful eating among adolescents in high-income countries: A mixed-methods systematic review. Obes. Rev..

