# Supplementary figures and images for: Risky Behaviors for Non-Communicable Diseases: Italian Adolescents’ Food Habits and Physical Activity
Source: Nutrients. 2024 Nov 30;16(23):4162. doi: 10.3390/nu16234162 (PMC11644692; doi:10.3390/nu16234162)

**Figure S1.** Distribution of correct dietary habits by sex: score from 0 to 5.

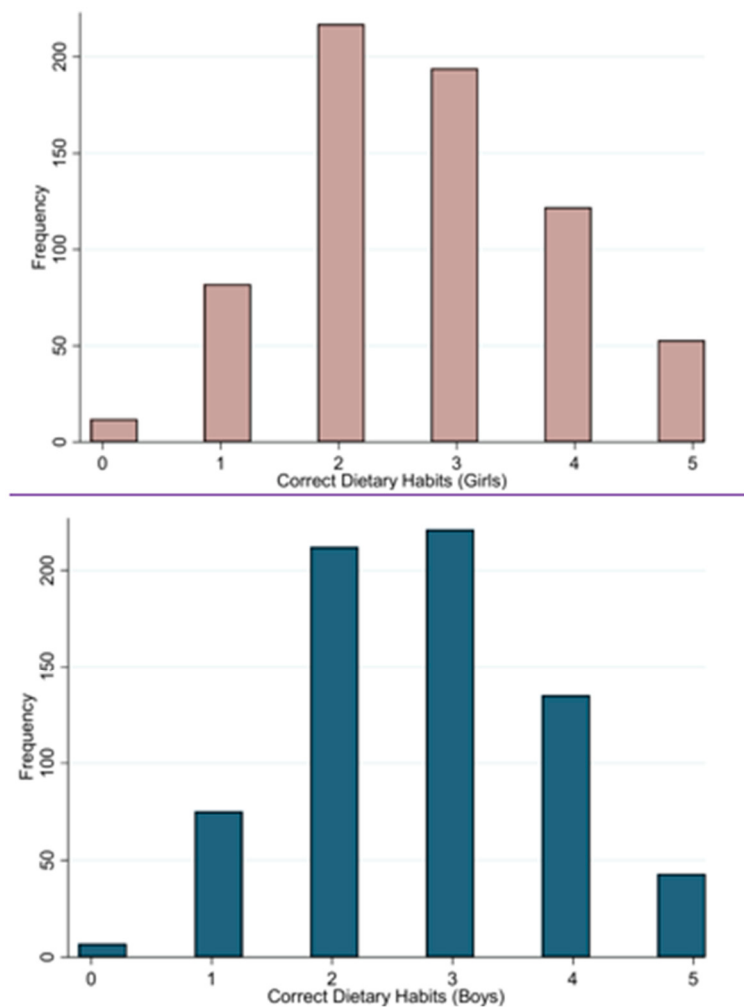

Supplement: Supplementary file 1 [file nutrients-16-04162-s001.zip › nutrients-3300651-supplementary.pdf]
